# Lung Cancer Patients’ Conceptualization of Care Coordination in Selected Public Health Facilities of KwaZulu-Natal, South Africa

**DOI:** 10.3390/ijerph192113871

**Published:** 2022-10-25

**Authors:** Buhle Lubuzo, Khumbulani W. Hlongwana, Themba G. Ginindza

**Affiliations:** 1 Discipline of Public Health Medicine, School of Nursing and Public Health, University of KwaZulu-Natal, Durban 4001, South Africa; 2 Cancer & Infectious Diseases Epidemiology Research Unit (CIDERU), College of Health Sciences, University of KwaZulu-Natal, Durban 4000, South Africa

**Keywords:** cancer care, coordination, communication, patients, low- and middle-income countries

## Abstract

Background: Cancer patients commonly receive care, including comprehensive treatment options, from multiple specialists within and across facilities offering varying levels of care. Given this multi-layered approach to cancer care, there is a need for coordinated care enhanced through integrated information flow for optimal patient care and improved health outcomes. Objective: This study aimed to explore how patients conceptualized cancer care coordination in an integrated health care system in KwaZulu-Natal. Methods: The study employed a grounded theory design to qualitatively explore the patients’ experiences and views on cancer care coordination using in-depth interviews. Guided by the grounded theory principles, data generation and analysis were conducted iteratively, followed by systematic thematic analysis to organize data, and review and interpret comprehensive findings. This process culminated in the development of themes relating to barriers to cancer care coordination and the interface between the primary and tertiary settings. Theoretical saturation was achieved at 21 in-depth interviews with consenting respondents. Results: This study revealed that care coordination was affected by multilevel challenges, including pertinent health system-level factors, such as difficulty accessing specialty care timeously, weak communication between patients and healthcare providers, and unmet needs concerning supportive care. We found that negative experiences with cancer care erode patient trust and receptiveness to cancer care, and patients advocated for better and proactive coordination amongst different care facilities, services, and providers. Conclusions: An integrated care coordination setup is essential to create and sustain a high-performance health care system. These findings make a case for developing, implementing, and evaluating interventions to enhance the quality of cancer care for patients and ultimately improve health outcomes for patients in KwaZulu-Natal. This study will provide comprehensive data to inform professionals, policymakers, and related decisionmakers to manage and improve cancer care coordination.

## 1. Study Background

A cancer diagnosis, often seen as a life-threatening condition, marks the start of a journey through which a patient enters the healthcare system and navigates an unfamiliar, frightening, and emotional experience towards an often-uncertain destination [1,2,3,4,5,6,7,8,9,10,11,12,13,14,15,16,17,18]. The health care system consists of disciplines, people, and processes that primarily aim to promote, restore, or maintain health [2,7,8,9,10,19,20,21,22,23,24,25]. As more clinicians become involved in patient care, communication across multiple clinical teams becomes increasingly complex, and care coordination can be jeopardized [21,24,26,27,28,29,30,31,32,33,34,35,36,37]. Communication during the care pathways molds cancer patients’ relationships with those who manage and support them on their cancer journey. Teamwork among various health providers is an important recipe for improving communication and care coordination [38].

Although it is evident that communication substantially impacts both the patient experience and their psychosocial outcomes, poor communication remains a significant problem facing cancer patients across the care spectrum [25,30,33,36,39,40,41]. An effective care plan to reduce cancer morbidity and mortality requires optimal interactions between patients, caregivers, clinicians, and multiple specialties [2,7,8,9,10,19,20,21,22,23,24]. Therefore, improving cancer care coordination is a priority area for service improvement. Not only is good care coordination essential to optimize patients’ experience, but it has also been shown to reduce the future need for supportive care and improve psychosocial outcomes in other settings [13,33,42,43,44]. Effective coordination of care between clinicians, services, and health sectors throughout the patient journey is fundamental to providing high-quality care [15,45,46,47,48,49,50,51,52,53]. However, quality improvement initiatives are hindered by the scarcity of accurate and reliable measures and/or tools for this aspect of cancer care. 

Research focusing on health care delivery has revealed the complexity of coordinating care for people with cancer and proposes some promising interventions [54,55]. Recent studies and reviews on cancer care coordination interventions have found that patient navigation, designated care coordinators, and collaborative care models are among the common approaches implemented [54,56,57,58,59,60,61,62]. Multiple barriers to improving the provision of cancer care exist, including lack of social support, financial concerns, and problems with healthcare communications; in this context, effective care coordination is required [8,12,24,63,64,65,66,67,68,69,70,71,72]. Coordination means synthesizing care goals and decisions across the multiple groups involved in patient care and mutually aligning, timing, and adapting critical care tasks among different care teams or team members over time [17]. Patients are ideally placed to share more profound insights into all the dimensions of cancer care coordination, as they are likely to be the only individuals present at every encounter with health services [70,72]. However, research on care coordination from the perspectives of patients is rare. Hence, the study aimed to explore the views of patients with lung cancer and their caregivers (as proxies) on the current state of cancer care coordination in KwaZulu-Natal (KZN) and explore key strategies to improve care. 

## 2. Methodology

### 2.1. Study Design

The study was undertaken using a grounded theory (GT) design rooted in qualitative research [73]. It is widely used in health and social sciences to generate theoretical accounts of social phenomena [74]. In-depth interviews were conducted with patients who had been diagnosed and/or treated for lung cancer or their family caregivers, as they were best placed to identify issues and share experiences of living with and receiving care for lung cancer from their perspectives, including the barriers, facilitators, and opportunities for improving care coordination. 

### 2.2. Study Settings

This study was conducted in the province of KZN, South Africa (SA). It was conducted among patients who received care at the oncology departments in the three public health facilities, namely Greys Hospital located in Pietermaritzburg (PMB) and Addington Hospital and Inkosi Albert Luthuli Central Hospital (IALCH), which are both located in Durban (DBN). DBN and PMB are located in eThekwini and uMgungundlovu District Municipalities, respectively, and are the two most populous districts in KZN Province. While PMB is the province’s capital city, DBN is the largest city in the province. 

SA has a two-tiered and highly unequal healthcare system: the public and private sector. The public sector is funded by the state and provides to the majority of the population. The private sector is largely funded through individual contributions to medical aid schemes or health insurance. In 2021, SA had a population of approximately 60 million people [75]. As such, the percentage of individuals who were not covered by a medical aid scheme was 83.9% with just 16.1% covered [75]. Nationally, KZN was one of the provinces that most commonly use public health facilities (78.7%) as only 10.5% of its population are members of a medical aid scheme [75]. 

The three health facilities are the only public hospitals offering oncology services in the province. These include an oncology department with radiotherapy and chemotherapy. In addition, IALCH receives nationwide referrals and has highly specialized services, including a pathology laboratory, hematology, and nuclear medicine. The public health service structure in SA and KZN province follows a pyramidal approach, organized in referral patterns that start from primary to secondary, tertiary, quaternary, and medical training institutions [76]. At the tertiary level are the three hospitals, where advanced diagnostic procedures, treatments, and supportive care are received. A patient in the public sector may be referred to a palliative care program by a healthcare worker at a hospital or referred to external institutions such as support groups and hospices. However, the current pathway is complex and tends to prolong the time for a patient to access the advanced services offered at tertiary levels [12,25,67]. Navigating these complex health care structures is difficult, thereby decreasing the likelihood of favorable health outcomes and patient satisfaction [25,47,67,72].

### 2.3. Study Participants and Recruiting Approach

The eligibility criteria included participants over 18 years of age who had been or are undergoing treatment for lung cancer and were cognitively able to participate. Eligible patients were identified through a list of patient names received at the oncology department and/or were identified during their visits to the participating facilities. The first author later contacted the potential participants to share details of the study and an invitation to participate. For patients who met the eligibility criteria and were willing but were too sick to participate, we used a modified version of snowballing [77] to identify their family caregivers, who acted as proxies. A proxy is a person authorized to act on behalf of someone else [78]. This process was carried out through the patients they cared for. All potential participants were contacted and provided with details of the study. Those who consented were subsequently contacted to arrange for an interview, and participants were not compensated.

### 2.4. Data Collection and Sampling

Twenty-one in-depth interviews were conducted by the first author and a research assistant, who are experienced in qualitative research methods, using a detailed semi-structured interview guide. The interviews were conducted using virtual (Zoom and telephonic) platforms. The use of virtual platforms was driven by the disruptions caused by the COVID-19 pandemic on how research is conducted [79]. The interview guide was developed using an iterative process, with the research team revising it several times and consulting the literature. In the interview guide were open-ended questions and probes to deepen the exploration of the phenomenon. Questions were organized into three domains: (1) a description of the experience of their care; (2) the management (coordination) of their care by health professionals; and (3) possible changes to the healthcare system to aid care coordination. 

All interviews were conducted in either isiZulu or English. Limited demographic data, including the level of education, were collected. Each interview lasted approximately 40 min and was conducted at a time suitable for the participant. Audio-recorded (with the permission of participants) and transcribed data were entered into NVivo 8 (qualitative data analysis) software [80] for analysis. Concurrent data generation and analysis are fundamental for general qualitative research and GT research design [81]. Therefore, data generation and analysis were conducted iteratively, guided by the principle of data saturation [68], whereby no new themes or meanings emerged. 

### 2.5. Data Analysis

Data collection and analysis took place concurrently, allowing each proceeding interview to be informed by those that preceded it [81]. The analysis was achieved through the use of transcripts. Transcripts for each participant were de-identified to ensure confidentiality and limit analytical bias among researchers, the first author, and a research assistant. The researchers reviewed the interview transcripts and searched for initial concepts and themes. In GT-based analysis, the researcher generally analyses the data through (a) finding repeating themes by thoroughly reviewing the data, (b) coding the emergent themes with keywords and phrases, (c) grouping the codes into concepts hierarchically, and then (d) categorizing the concepts through relationship identification [82,83,84,85]. 

Theoretical sensitivity encompassed the entire research process; it is the ability to know when one identifies a data segment that is important to one’s theory, thereby directing the ongoing data generation [86]. The construction process of these thematic categories and coding was inductive and deductive because the development of themes and subthemes rests on literature and emerging categories of empirical analysis [74,81,82,84,86,87,88,89,90]. Ongoing analysis and recruitment continued until saturation of themes was reached. Lastly, themes were developed and discussed by all the authors to reach consensus. Direct quotes from participants support interpretations of the findings.

## 3. Results

### 3.1. Respondents’ Characteristics

Sixteen patients with lung cancer and five proxies (caregivers) participated in the study. All respondents were unemployed, and nearly one-third (29%) were pensioners. Approximately 90% of respondents were Black, 19% were women, and almost half (48%) reported residing in a township. Most (76%) patients had received chemotherapy, and one refused treatment. Sixty-two percent had stage IV lung cancer, and the remainder were unsure of the staging. Respondents’ socioeconomic demographics and health-related characteristics are summarized in Table 1.

All interviews were conducted between May 2021 and April 2022. Five themes emerged from the analysis, namely (I) lack of integrated systems of care; (II) lack of care providers’ engagement with patients; (III) unclear health professional roles and responsibilities; (IV) unmet supportive care needs; and (V) opportunities for improved care coordination. These themes were interconnected.

### 3.2. Lack of Integrated Systems of Care

Care coordination ensures that patients’ needs are met, and integrated services are provided. Yet, patients and caregivers expressed concerns about timing and coordination and reported consistent difficulty with scheduling appointments and obtaining comprehensive referrals for an easy, fast, and safe transition between systems (respondents 2, 3, 5, 9, 11, 14, 19, and 21). As such, patients who received treatment, which is usually in different healthcare settings, reported a lack of effective referral between these settings, particularly from specialty care (tertiary) back to the local services (primary care) from which patients sought support services (respondents 1–4, 7–10, and 12–21). Nearly all the respondents expressed concern about communication being fragile during patient transitions across care settings. As a result, the most common views on the care delivery were apparent delays in diagnosis and/or treatment of cancer, as asserted by the participants below:

*Yes, there were delays in my treatment, mama [ma’am], and the process of care caused the problems that caused delays; there is a system used to attend to patients. They don’t attend to you even if you are sick to death because you need to go through their referral and booking system, which fails to work most of the time*.

*(Respondent 11)*

*They [healthcare providers] were clueless because they all started from scratch with their search. Everyone started from the beginning because no one knew anything about his condition. Even the referral letter was just blank; we saw it. The only written thing was that this patient is referred to hospital B and from there to hospital C for a scan, but nothing says to scan for what and what they managed to find. When we got to hospital C, it was the same thing; in fact, that day they even delayed admitting him because they didn’t know the patient was going to be admitted for what.*

*(Respondent 21: Proxy)*

In support of the sentiments conveyed by respondents 11 and 21, two patients dejectedly shared the following:

*Perhaps because I have cancer, I don’t know, maybe they are not saying, perhaps they are thinking I’m supposed to be dead or something like that, I don’t know; perhaps that’s why they’re pushing the dates back so far, and my care is delayed.*

*(Respondent 2)*

*The hospitals are overwhelmed with the numbers, and I believe it burdened the caretakers, nurses, maybe including the doctors. As a result, they see us now as numbers rather than as humans; that’s where you’ll see the patients complain. So maybe those are the things that may need to be addressed.*

*(Respondent 2)*

Most patients expressed general satisfaction with their specialty care team (respondents 2, 6, 7, 8, 9, 10, 12, 13, 14, and 18). However, some did not feel the primary care team had been very involved and/or supportive in their cancer care journey (respondents 1, 2, 4, 7, 9, 14, and 21). Overall, experiences ranged from almost no involvement to very little input and participation from the primary care team. As a result, patients considered that they were not knowledgeable on how to support their treatment and survivorship care (respondents 1, 4, 8–10, 18, and 21). 

In stark contrast, only 2 of 21 patients interviewed highlighted optimistic features of the care delivery:

*One thing I realized is that the public hospitals are overloaded with patients, but every doctor that I met was respectful, and the nurses were respectful. I can’t say they did anything; I would not be happy with my father, whom I was taking care of.*

*(Respondent 18: Proxy)*

*I would say they worked well together because I feel everything is going well, and if I happen to miss my appointment date, they can accommodate me. They phone to organize a new date, which shows that the doctors are working well together.*

*(Respondent 6)*

Manifesting in the patients’ views above was the extent to which patients value respect, especially expressions of concern, care, and empathy (respondents 6, 8, 14, 17, and 18). One patient remarked the following:

*But in terms of the attitudes, honestly, I can’t complain about the nurses. I can’t complain about the doctors; everyone was respectful. I wish they could keep up with that because you know what impresses the patients is not whether they get medication or not sometimes; it’s just the treatment. If they don’t respect the patient, that itself can kill the patient.*

*(Respondent 14)*

### 3.3. Lack of Care Providers’ Engagement with Patients

Views regarding communication varied widely and cut across barriers, with some patients expressing challenges with communication with providers, whereas others had favorable experiences. Effective information exchange and a positive interpersonal relationship with the clinical team were of fundamental importance to patients and family members (respondents 1–4, 7–10, and 12–21). The trusted rapport with a provider was thought to have enabled them to make sense of the information they received during their care.

*When my husband was admitted to hospital A, the biggest problem was that they did not explain what was going on. This one time, they made us wait up to 3 weeks, just waiting to do a scan, and for that three weeks, they were not updating us as to what was going on, and we couldn’t visit him in hospital because it was COVID days… Now when doctors come, they come with students. They don’t explain anything to patients, but their students and students also don’t explain anything to patients, and my husband was admitted for three weeks with no information; they kept saying he needed to do some tests but then not specific about what tests, so that was very disappointing.*

*(Respondent 16: Proxy)*

*I don’t know what was wrong with hospital A; I told you, we tried many times to communicate with the doctor there and even asked the nurse if they could give us even just 2 min as a family; we needed answers, but it was hard.*

*(Respondent 4)*

*I would say that available health workers do their job well, but I wouldn’t know their communication because the challenge with the big doctors that come to the wards, we couldn’t communicate with them and ask them questions; we were not given that chance even though there was this one particular doctor that we would raise our concerns with, but you would see that he does not pay attention; he has no time for that.*

*(Respondent 14)*

In instances where communication existed, some patients referred to difficulty absorbing information immediately after their diagnosis, as their thought processing was still weakened by the stress of their confirmed cancer diagnosis that, as such, makes one’s symptoms feel worse (respondents 3, 4, 11, and 17); one patient echoed the following:

*When told of my diagnosis, I was confused at first after a long period of not knowing what it was. I did not accept it quite well because of how they informed me. It was as if I would die soon, the manner of approach was not good, but I still survived in those hopeless conditions.*

*(Respondent 17)*

Additionally, the most urgent problem in the comprehensiveness of information shared during diagnosis was patients’ uncertainty about the implications of cancer. Patients and caregivers repeatedly reported inadequate comprehension of their prognosis (respondents 3, 5, 11, 14, 16, and 19). Respondents agreed that they still did not have detailed knowledge about their illness even after being diagnosed and undergoing treatments. Examples of these events include insufficient information provided to the patient, as resonated by one patient:

*I really wish to get more information because I do not have any experience with cancer. It was the first time when I was diagnosed.*

*(Respondent 14)*

Even when communications were considered generally respectful and professional, a number of the negative experiences reported by the study respondents related to what they perceived as a failure to recognize them as unique individuals, different from any other patient, and to tailor care approaches accordingly (respondents 5, 11, 14, 19, and 20).

### 3.4. Unclear Health Professional Roles and Responsibilities

Cancer patients tend to have multiple interactions with the healthcare system and get to see several different specialists in different care facilities. In most cases, they strikingly lack one provider who can oversee and coordinate clinical results and follow patients in the course of their care (respondents 3, 5, 11, 15–17, 20, and 21). One patient stated,

*I wouldn’t say much because we would get different doctors whenever we went to hospital A. After all, whenever we went there, we would find maybe another doctor at that particular time.*

*(Respondent 15)*

As a result of multiple interactions, several respondents acknowledged a mix-up in the roles and responsibilities of the different members of the healthcare team involved in their care (respondents 4, 5, 11, 13, 15, 17, 18, and 21). When sharing their care experiences, many patients and caregivers asserted that multiple healthcare workers were involved in their care, thereby making it difficult to remember exactly who did what or who was responsible for which aspect of their care (respondents 4, 5, 11, 13, 15, 17, and 21), as captured below:

*I will be telling lies if I say there was someone who explained which doctor was going to do what and which doctor would do what. There you find doctors doing their rounds, coming to you as a team, and one doctor would explain to the rest of the team about your condition for them to know but not that they discuss with me as a patient.*

*(Respondent 5)*

Moreover, patients did express some uncertainty over the role that primary care could play in their cancer care (respondents 1, 2, 4, 7, 9, 14, and 21). They were unsure whether their primary care team would have the necessary expertise (respondents 1, 4, 8–10, 18, and 21).

### 3.5. Unmet Supportive Care Needs

Participants were congruent when asked what they understood about their care coordination, saying care coordination must focus on more than just clinical services. The most frequently reported unmet needs for coordination were those in the activities of the daily living domain, such as home or self-care and financial needs, followed by access to information and psychosocial needs (respondents 1–4, 7–10, 12, 13, and 15–21). Respondents focused mainly on physical barriers to care that influenced adherence to treatment plans (respondents 1–5, 7–12, 14, and 16–21). The location of large specialist centers primarily in urban areas presents physical and economic challenges to patients residing in remote areas, including distance, limited access to transport, and the related cost of travel (respondents 1–4, 7–10, 12, 13, and 15–21).

Practical measures such as assistance with transportation throughout the cancer care experience, as a result, were also seen as an indispensable fragment of supportive care (respondents 1–5, 7–12, 14, and 16–21). Two patients shared the following:

*I am forced to wake up very early at midnight so that I can prepare and get ready to catch the first taxi by 04:30 a.m. because the hospital can get packed. We live far from the hospitals, so we are forced to wake up so early in the dark to catch public transport. From home, I take a taxi to town, and there again I take another taxi to the hospital, and when you get there, you hold the queue. Sometimes the appointment date comes when I don’t have money for transport, I would then knock door to door, borrowing transport money from neighbors to manage to save my life.*

*(Respondent 12)*

*Hospital C told me to phone them whenever I face any difficulties, but I can’t even phone them because I usually don’t have airtime. I would have called them to report that my burning, swollen feet and bones can be so painful as if someone had been lashing me. So, I just report all the matters when I get there on my appointment date, but I cannot call because of airtime.*

*(Respondent 10)*

In contrast, one patient presented good feedback on getting assistance from a doctor concerning his financial challenges. He stated, “I am now sorted; the doctor helped me get pension money” (Respondent 15).

### 3.6. Opportunities for Improved Care Coordination

The respondents offered suggestions for improving cancer care coordination. They advocated for better and proactive coordination amongst different care facilities, services, and providers (respondents 1–4, 7–10, and 12–21). Flexibility in providing care and practical support were also identified to be critical in increasing service accessibility.

Most respondents proposed that a case manager (contact person) position is established to ensure care coordination and communication (respondents 1–5, 8, 9, 11–14, and 16–21). Participants viewed nurses or social workers as appropriate for serving as the contact person or navigator:

*The patient would still be shocked that he is diagnosed with cancer, and cancer is a severe illness, despite the availability of chemo and radiation therapy. However, you are still under the shock that you might die soon. So, I would advise that they [the healthcare providers] allocate a nurse or someone who will stimulate the mindset of the patients and give them hope that even though they are cancer patients, it does not mean that they will die soon.*

*(Respondent 12)*

*There wasn’t any specific individual that we would say has been assigned to him [patient] that will be a coordinator between ourselves and them [healthcare providers] whenever we go there [healthcare facilities]. Even that could be a good idea if a patient could have a contact person that you know when you have this, you could call.*

*(Respondent 20: Proxy)*

*So that whenever I see something that might be developing, I would be able to contact that person for awareness even before the appointment date.*

*(Respondent 8)*

Moreover, patients and caregivers suggested that cancer care delivery are brought closer to their homes and communities (respondents 1–5, 9, 12, 15, and 21). For example, they encouraged introducing home-based services to address current care-delivery challenges.

*Mainly, if we could have community support groups, which maybe could be led by retired nurses, other people maybe are retired who could be trained to understand what kind of questions and how are cancer patients different from other patients, be trained to even visit a family once in a while.*

*(Respondent 9)*

*I want to emphasize the need for a social worker, because this [cancer] is between life and death, and I think they should have them on offer while it is still early. Understand that when the patient is diagnosed, they need that support, because so many things happen, so it should be readily available. My father still needs that support service, and we are struggling. They did not link us with anything or anyone.*

*(Respondent 3: Proxy)*

**Figure 1:**
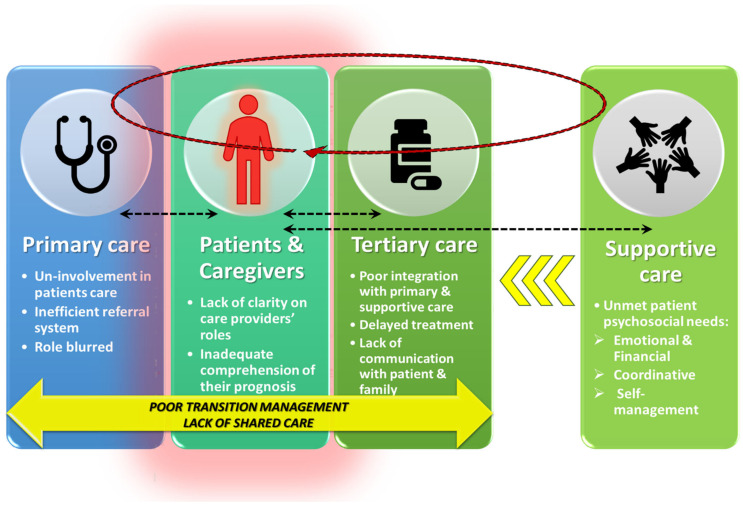
Summary of the key findings derived from this study.

Finally, respondents were of the view that, if their suggestions were implemented, they would provide patients with necessary hope (respondents 1–4, 6–9, 11, 13, and 16–20). In support, most patients appeared to have welcomed a call or other proactive contact from the healthcare team at the time of diagnosis or initial treatment. Fear of a cancer diagnosis was an overarching issue that affected one’s coping mechanism. Therefore, respondents agreed that the healthcare team needed to be willing to answer questions and provide information and emotional support to patients and their families. By this, the patient is empowered, and there might be an improvement in how they feel and even their life experience with cancer. The findings presented in this paper can be summarized through the following illustrative diagram below (Figure 1).

The diagram summarizes the multilevel challenges highlighted by the patients and what the study brings forward as possible solutions to coordinated care through arrows.

## 4. Discussion

Improving care coordination has numerous benefits, including short-term and long-term improved patient care. Studies from the patient perspective have documented that effective communication with health care providers is a high-priority concern throughout the cancer trajectory [9,72,91,92,93]. Nevertheless, in this study, cancer patients and their caregivers reiterated central issues related to a lack of communication, lack of information exchange by the healthcare providers, and disturbed care continuity.

Effective health care delivery depends primarily on the interaction between health care providers, those seeking care, and their loved ones [91,94]. However, our study results suggest that care coordination and communication problems are most intense at the interface between healthcare settings and between care providers and patients. The complexity of cancer care suggests that efforts to improve coordination and communication must be multilevel, acknowledging and addressing patient, clinician, organizational, and policy barriers and facilitators [93,95]. We found that when referred back to primary care for ongoing disease management, patients with cancer lacked a consistent and timely connection to primary care for further support, and this hindered the continuity of their care. This suggests a need for care-delivery strategies that link oncology and primary care by enhancing communication and outlining roles and responsibilities across healthcare providers. With communication being the most vital factor, patients believe that providing appropriate and adequate information will empower them to engage and be expert patients in their care.

We found that negative experiences with cancer care erode patient trust and receptiveness to cancer care. Strategies that enhance communication and coordination of care may help equip the patients and their caregivers with knowledge/information, further reducing incorrect assumptions and making cancer care more efficient [15,38,93,95], as respondents also suggested. Failure to narrow the gaps in the interface of cancer care may contribute to ongoing anxiety for patients, confusion about the healthcare team’s roles and prognosis, and delayed supportive care. Significantly, systems for coordinating cancer care and helping patients navigate the complex healthcare system must ensure that patient outcomes are maintained or improved [15,96]. A cancer diagnosis marks the start of a long journey, and the need for patients to embark on such a complex journey is hardly ever embraced. In relation to socioeconomically disadvantaged patients, our findings are consistent with extant research that shows cancer patients who are lower-income are more likely to report suboptimal communication and inadequate follow-up care and monitoring [68,72,97,98]. This may also be resulting in the financial constraints and needs indicated by the participants in relation to transportation costs and self-management care. This creates barriers to a seamless link between health care sectors and results in disconnection of care. The recommended strategies provided by respondents in this study align with several World Health Organization (WHO)-identified actionable priorities to enhance continuity and coordination of care [99]. The concept of a patient navigator should be considered [56]. The navigator may also assist with monitoring and facilitating the patient’s care [100,101,102,103].

We aimed to identify the challenges posed to patients and caregivers by a cancer diagnosis and suggest various ways these might be best met. Our findings show that a better understanding of patients’ views of care coordination can inform ways to improve cancer care delivery. Such information may benefit providers, hospitals, policymakers, and patients as we develop effective and efficient care coordination models. Given the increasing importance of providing care for patients with complex diseases and chronic conditions, our findings may rekindle ideas about efficient strategies and interventions to reduce care coordination problems that often lead to lower-quality care. This analysis was strengthened by its grounded theory approach. Iterative data collection and analysis facilitated the interpretation of respondents’ experiences. 

Despite these strengths, the study has several significant limitations, including using virtual platforms to conduct the interviews as a result of the COVID-19 pandemic. Although the approach offered a comfortable, non-intrusive, safe, convenient, and easy setup, there were limitations related to lack of non-verbal communication and poor interaction between interviewer and participants, as conversations would have possibly flowed better face-to-face. As a result of having conducted this study during this pandemic, this might have heightened some of the identified barriers to coordinated care in this study. However, only a few participants attributed their concerns to the pandemic. Whilst the true impact of the possible disruptions is a significant aspect to discover, the nature of our study did not openly explore this impact. Nevertheless, strategies should be considered to minimize possible interruptions to cancer care. We identified care coordination challenges among lung cancer patients at the tertiary care level. More diverse patient enrollment (at various levels of care) in future research is necessary. Regardless of these limitations, our findings have important implications; as policymakers search for ways to transform our cancer care-delivery system, incorporating patients’ perspectives is critical to ensure that patients and their needs inform the efforts to improve care coordination. The identification of current obstacles has the potential to guide the development of future initiatives to improve the quality of coordinated health care.

## 5. Conclusions

Care coordination is essential to facilitating and supporting high-quality cancer care. This qualitative study identified multilevel challenges to care coordination. We conclude that an integrated care coordination setup is essential to create and sustain a high-performance health care system. These are assessed by how the patients and their families that receive the services experience the provision of care. Even when a successful treatment is received, the effects of having had cancer can be long-term, resulting in a need for continuity of supportive care. We hope the views described here will stimulate new funding and research, ultimately improving cancer care quality for all patients.

## Figures and Tables

**Table 1:** Characteristics of respondents (N = 21).

#	Sex	Age	Type of Participant	Marital Status	Educational Level	Employment Status	Geographic Location	Disease Staging	Treatment (Tx)
1	F	70s	Proxy	Widowed	High school	Pensioner	Urban	4	Refused Tx
2	M	70s	Patient	Married	Primary school	Pensioner	Urban	4	Chemotherapy
3	M	30s	Proxy	Single	High school	Unemployed	Township	-	Chemotherapy
4	M	60s	Patient	Married	Secondary school	Unemployed	Township	4	Chemotherapy
5	M	60s	Patient	Single	No school	Unemployed	Township	4	Not initiated
6	M	70s	Patient	Married	High school	Pensioner	Township	4	Not initiated
7	M	60s	Patient	Married	Primary school	Unemployed	Township	4	Chemotherapy
8	M	60s	Patient	Married	High school	Unemployed	Township	-	Chemotherapy
9	M	60s	Patient	Married	Not shared	Unemployed	Township	-	Chemotherapy
10	M	50s	Patient	Married	Primary school	Unemployed	Township	-	Chemotherapy
11	M	30s	Patient	Single	Primary school	Unemployed	Township	4	Chemotherapy
12	M	50s	Patient	Married	High school	Unemployed	Rural	-	Chemotherapy
13	M	60s	Patient	Single	Secondary school	Pensioner	Township	-	Chemotherapy
14	M	30s	Patient	Single	High school	Unemployed	Rural	-	Chemotherapy
15	M	50s	Patient	Married	Not shared	Unemployed	Urban	4	Chemotherapy
16	M	70s	Proxy	Married	Tertiary	Retired	Urban	-	Chemotherapy
17	F	20s	Patient	Single	High school	Unemployed	Rural	4	Chemotherapy
18	M	40s	Proxy	Single	Primary school	Unemployed	Rural	4	Chemotherapy
19	M	60s	Patient	Married	High school	Pensioner	Urban	4	Chemotherapy
20	F	30s	Proxy	Single	Tertiary	Unemployed	Rural	4	Not sure
21	F	40s	Proxy	Widowed	High school	Unemployed	Township	4	Chemotherapy

## Data Availability

Data from this study are the property of the KwaZulu-Natal Department of Health and UKZN and cannot be made publicly available. All interested readers can access the data set from the UKZN Biomedical Research Ethics Committee (BREC) from the following contacts: The Chairperson Biomedical Research Ethics Administration Research Office, Westville Campus, Govan Mbeki Building UKZN P/Bag X54001, Durban, 4000 KZN, SA, Email: BREC@ukzn.ac.za; Tel.: +27-31-260-4769; Fax: +27-31-260-4609.

## Abbreviations

BMSF:	Bristol–Myers Squibb Foundation
CIDERU:	Cancer & Infectious Diseases Epidemiology Research Unit
DBN:	Durban
GT:	Grounded theory
HICs:	High-income countries
IALCH:	Inkosi Albert Luthuli Central Hospital
KZN:	KwaZulu-Natal
LMICs:	Low- and middle-income counties
MLCCP:	Multinational Lung Cancer Control Programme
PMB:	Pietermaritzburg
SA:	South Africa
UKZN:	University of KwaZulu-Natal
WHO:	World Health Organization
